# Genome-wide analysis of the effect of histone modifications on the coexpression of neighboring genes in *Saccharomyces cerevisiae*

**DOI:** 10.1186/1471-2164-11-550

**Published:** 2010-10-09

**Authors:** Yangyang Deng, Xianhua Dai, Qian Xiang, Zhiming Dai, Caisheng He, Jiang Wang, Jihua Feng

**Affiliations:** 1 School of Information Science and Technology, Sun Yat-Sen University, 135 West Xin'gang Road, Guangzhou, PR China

## Abstract

**Background:**

Neighboring gene pairs in the genome of *Saccharomyces cerevisiae *have a tendency to be expressed at the same time. The distribution of histone modifications along chromatin fibers is suggested to be an important mechanism responsible for such coexpression. However, the extent of the contribution of histone modifications to the coexpression of neighboring genes is unclear.

**Results:**

We investigated the similarity of histone modification between neighboring genes using autocorrelation analysis and composite profiles. Our analysis showed that neighboring genes had similar levels or changes of histone modifications, especially those transcribed in the same direction. The similarities, however, were restricted to 1 or 2 neighboring genes. Moreover, the expression of a gene was significantly correlated with histone modification of its neighboring gene(s), but this was limited to only 1 or 2 neighbors. Using a hidden Markov model (HMM), we found more than 2000 chromatin domains with similar acetylation changes as the cultures changed and a considerable number of these domains covered 2-4 genes. Gene pairs within domains exhibited a higher level of coexpression than random pairs and shared similar functions.

**Conclusions:**

The results of this study suggest that similar histone modifications occur within only a small local chromatin region in yeast. The modifications generally have an effect on coexpression with only 1 or 2 neighboring genes. Some blocking mechanism(s) might strictly restrain the distribution of histone modifications in yeast.

## Background

Genes are not distributed randomly within a genome [1-3]; in mammals, housekeeping genes and tissue-specific genes show a strong tendency to be clustered together [4,5] and genes that participate in the same biochemical pathway tend to be located close together in the genome [1,6,7]. Moreover, similar expression levels or expression patterns have been found in neighboring gene pairs in various eukaryotic genomes [1,6,7].

The coexpression sharing similar regulatory elements and chromatin environment have been proposed as two major factors responsible for coexpression of neighboring genes but the underlying mechanism is unclear [1,8,9]. During transcription, transcription factors (TFs) regulate gene expression by binding to the *cis*-regulatory elements. Neighboring genes have more chance to share promoters or regulatory elements and to be coregulated and coexpressed [10,11]. Alternatively, factors in the chromatin environment, such as histone modification, nucleosomes etc., can modulate gene expression within a local region. Neighboring genes are more likely to be located within the same chromatin domain and to share a similar chromatin status, thus having a greater likelihood of coexpression than non-neighboring genes [1,9,11,12].

Neighboring gene pairs in the budding yeast *Saccharomyces cerevisiae *exhibit stronger coexpression than random pairs [10]. Sharing 5' regulatory elements was suggested to result in the coexpression [11]; however, the results of later studies disagreed with this hypothesis. Whole-genome expression data analysis has shown that divergent pairs (when the transcriptional directions of immediate neighboring pairs are divergent, i.e. ← →) do not exhibit significantly higher levels of coexpression than parallel pairs (← ←/→ →), although divergent pairs are more likely to share the same regulatory system [10]. Moreover, from an evolutionary perspective, Tsai *et al. *[13] reported that adjacent pairs with a shared transcription factor-binding site (TFBS) have no higher rate of coexpression than those without. That result was confirmed by Batada *et al. *[12], who showed that when the similarity of TF regulation between gene pairs is controlled at the same level, adjacent gene pairs have much higher coexpression rates than unlinked pairs. All of these findings taken together indicate that the commonly shared *cis*-regulatory system cannot solely account for the coexpression of adjacent gene pairs in yeast. It is strongly suggested that other mechanisms, e.g. regulation at the chromatin level, might play a more important role in the coexpression of neighboring pairs of genes [12].

Several models have been proposed to explain how chromatin regulation affects coexpression in neighboring genes [1,9,12,14]. According to the model proposed here, histone-modifying enzymes might be propagated along the chromatin fiber and form an extended domain. Neighboring genes within a domain share the same molecular environment and thus might be coexpressed [1,14].

A localized distribution of silencing-associated histone modifications, such as H3K9me and H3K27me, has been found in fission yeast (*Schizosaccharomyces pombe*), fly (*Drosophila melanogaster*) and in mammals [14,15]. In budding yeast (*Saccharomyces cerevisiae*), SIR proteins (deacetylases) might be clustered in chromatin and mediate the formation of silent chromatin [16] and the Hda1-deacetylated domains have been observed in subtelomeric regions [17]. Furthermore, histone deacetylation sites have been reported to serve as possible partitions of chromatin domains in yeast [18]. Besides silencing, a range of modifications associated with activation has been reported for some individual genes. For example, acetylation of H3 and methylation of H3K4 can diffuse to the neighboring transgenic promoters in a transgenic experiment with human cells [19]. In *Aspergillus parasiticus*, acetylation of H4 can occur continuously and regulate the activation of genes within the aflatoxin cluster [20].

In the yeast genome, the vast majority of genes are generally in an active or potentially active state, and most chromatin regions are in an open or half-open status and marked by the activation-associated histone modifications, such as H3K9ac, H3K4ac, H3K4me3 etc. These modifications are highly associated with transcriptional activity [21,22]; therefore, the activation-associated modifications in yeast might play an extensive and predominant role in regulation of the chromatin level.

It is not known whether clustering of modifications is prevalent or to what extent this affects the coexpression of neighboring genes. To address this issue, we systematically investigated the relationship between the coexpression and co-modification of neighboring genes in *S. cerevisiae*. This study has provided clear evidence that activation-associated histone modifications have an effect on the coexpression of neighboring genes; however, the effect can be limited to only 1 or 2 neighboring genes.

## Results

### Co-modifications occur in 1 or 2 neighboring genes

The degree of co-modification between neighboring genes was measured by the autocorrelation coefficient, which is a general concept in signal processing and describes the degree of similarity between a given time series and a lagged version of itself over successive time intervals. The degree of autocorrelation was calculated for each chromatin region that included an equal number of genes (the regions were allowed to overlap but had at least 1 different gene) in the whole genome. The averaged autocorrelation over all regions was used to estimate the extent of co-modification (Methods).

**Figure 1:**
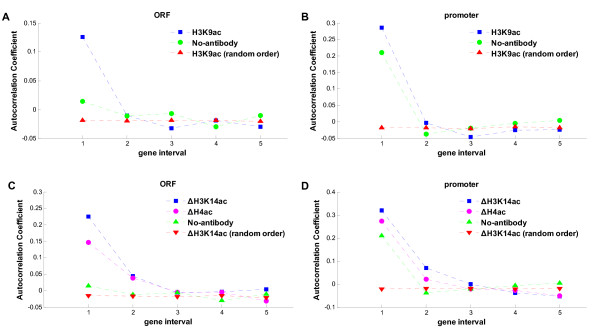
**The mean autocorrelation within 5 gene intervals**. The autocorrelation of histone modification was calculated for 50 linked genes. The mean of autocorrelation within 5 gene intervals is shown. The autocorrelation of random order (the order of genes was randomized) and of the control data (no-antibody) are shown as the controls. (A) Mean autocorrelation of H3K9ac in translated regions (ORF). The modification level of a gene was defined as the mean occupancy in its translated region. (B) Mean autocorrelation of H3K9ac in promoter regions. The modification level of a gene was defined as the mean occupancy in its promoter region. (C) Mean autocorrelation of ΔH3K14ac and ΔH4ac in translated regions. (D) Mean autocorrelation of ΔH3K14ac and ΔH4ac in promoter regions.

Figure 1A and 1B are plots of the mean autocorrelation of H3K9ac and show clearly that the immediate neighboring genes (gene interval = 1) had a significantly higher autocorrelation than random genes (the arrangement of genes within each region was randomized) in both gene translated and promoter regions (*p *< 10^-307 ^(Methods), Wilcoxon rank sum test). A high degree of autocorrelation was observed also in neighboring genes with 1 gene between them (gene interval = 2, translated region, *p *< 0.001; promoter region, *p *< 10^-11 ^Wilcoxon rank sum test). There was no significant degree of co-modification in genes further apart.

To test whether there was a systemic bias inducing co-modification, we computed the autocorrelation of the no-antibody control data. As shown in Figure 1A and 1B, the control data had a greater degree of autocorrelation than random in immediate neighboring genes (*p *< 10^-307^, Wilcoxon rank sum test, for both translated and promoter regions). However, the autocorrelation was significantly lower than that of H3K9ac (*p *< 10^-307^, Wilcoxon rank sum test), which means that although there could be a systemic bias, it is not the main source of co-modification. There was no significant difference of the degree of autocorrelation between H3K9ac and control data in the neighboring genes with 1 gene between (*p *> 0.01, Wilcoxon rank sum test), which indicates that the co-modification in neighboring genes with 1 gene between could be caused by the systemic bias. Similar results were observed for H3K14ac, H4ac, H3K4me2, H3K4me3 and H3K79me3 (Additional file 1, Figure S1A-D) but not for H3K4me1 or H3K36me3 (Additional file 1, Figure S1E and F).

We applied the autocorrelation analysis to the change of H3K14ac (ΔH3K14ac) and H4ac (ΔH4ac) when the cultures were changed from YPD to hyperoxic conditions. The higher degree of autocorrelation was found in the immediate neighboring genes and in neighbors with 1 gene between (Figure 1C and 1D, *p *< 10^-307^, for all comparisons with random or control data, Wilcoxon rank sum test). When the gene interval was >3, however, there was no significantly higher degree of autocorrelation; i.e. genes showed similar changes of histone acetylation only with 1 or 2 neighbors.

**Figure 2:**
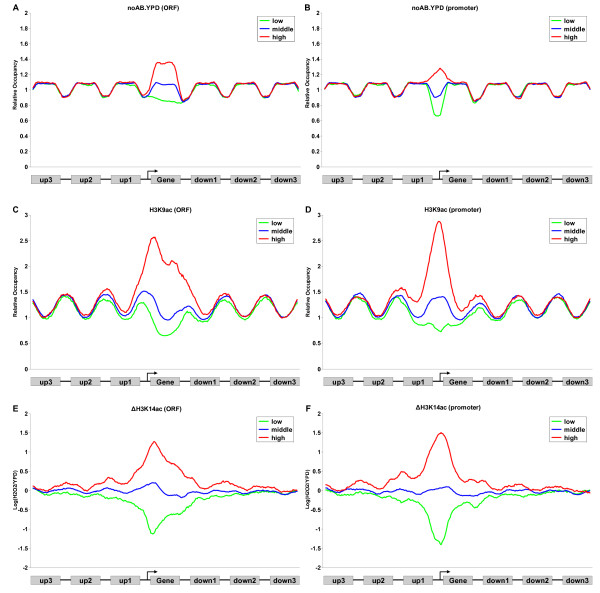
**The composite profiles on an average gene and its neighbors**. Composite profiles on 7 linked genes according to the occupancy or change of histone modification of the target gene (the middle one) observed. The target genes were assigned to I of 3 groups according to their mean level of occupancy or change in translated or promoter regions (low, bottom 20%; middle, middle 60%; and high, top 20%). The ends of ORFs were fixed and each translated region and intergenic region was divided equally into 40 and 20 bins, respectively. The average occupancy or change of modification for each bin is plotted. (A) and (B) Composite profiles of no-antibody control data according to the data level in translated/promoter regions of the target gene. (C) and (D) Composite profiles of H3K9ac according to the H3K9ac level in translated/promoter regions of target gene. (E) and (F) Composite profiles of ΔH3K14ac according to the ΔH3K14ac level in translated/promoter regions of the target gene.

We used the composite profiles to further illustrate similar histone modifications of neighboring genes to show the co-modification more clearly. The composite profiles revealed the similarity of modifications by displaying different distributions of modifications in a local region (Methods). In contrast, the composite profiles of the control data did not show any similarity in neighboring regions (Figure 2A and 2B, for translated and promoter regions, respectively). However, H3K9ac and ΔH3K14ac showed a clear similarity between the observed gene and its neighboring regions (Figure 2C-F). Similar results were observed for H3K4ac, H4ac, H3K4me2, H3K4me3, H3K79me3 (Additional file 1, Figure S2A-J) and ΔH4ac (Additional file 1, Figure S2Q and P), but not for H3K36me3 or H3K4me1 (Additional file 1, Figure S2K-N). These results further confirm the findings obtained from the autocorrelation analysis described above.

The change of acetylation exhibited a more significant similarity than the acetylation occupancy within neighboring 1 and 2 genes (Additional file 1, Figure S3). We suspected that the dynamic status of the histone acetylation might occur also in the neighboring regions more easily than the static status. The work described in what follows was focused on the change of acetylation instead of the occupancy and we designate a similar change of acetylation as coacetylation.

### The coacetylation of neighboring genes is associated with gene directions and distances

**Figure 3:**
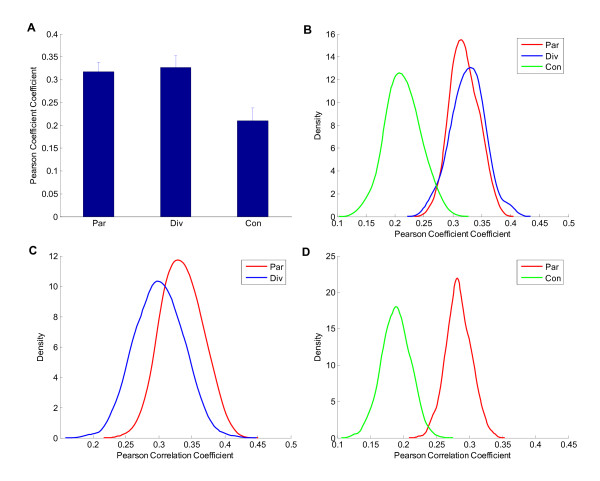
**Comparing the correlation of ΔH3K14ac between immediate neighboring genes of different directions**. The correlation was calculated by a bootstrap analysis (repeated 1000 times) across all neighboring parallel (→ →), divergent (← →) and convergent (→ ←) pairs. The ΔH3K14ac level of a gene was defined as the mean level of ΔH3K14ac in its translated region. (A) Mean correlation of gene pairs with different transcriptional directions (Par, parallel pairs; Div, divergent pairs; Con, convergent pairs). The error bar is the standard deviation. (B) Distribution of correlation for divergent (red), parallel (blue) and convergent (green) gene pairs. (C) Distribution of correlation for parallel (red) and divergent (blue) pairs whose ORF 5' distance was 600~1200 bp. (D) Distribution of correlation of parallel (red) and convergent (green) pairs whose ORF 5' distance was 1600~3600 bp.

Genes in a genome can be transcribed in one of two directions and therefore pairs of genes can be orientated in one of three alternative combinations; divergent transcription (← →), parallel transcription (→ →/← ←) or convergent transcription (→ ←). To test whether the gene directions of neighboring pairs had an effect on co-modification, we assigned the immediate neighboring gene pairs to divergent, parallel or convergent group according to their transcribed directions. Here, co-modification was defined as the Pearson correlation coefficient between gene modifications across gene pairs within the group, and we used a bootstrap analysis to compute it. The results are shown in Figure 3A and 3B, where it can be seen that co-modification of ΔH3K14ac in the parallel and the divergent groups was very close, but the convergent group had significantly lower co-modification than the other two (*p *< 10^-307^, Wilcoxon rank sum test).

We found that the 5' distances of the gene groups described above were significantly different (*p *< 10^-307^, Wilcoxon rank sum test, Additional file 1, Figure S4), and we speculated that the difference of coacetylation was due mainly to the gene separation distance. When we removed the gene pairs with too short or too long a distance between them and the 5' distances of the groups were almost equal, coacetylation in the parallel group was significantly higher than that in the divergent group or the convergent group (*p *< 10^-307^, Wilcoxon rank sum test, Figure 3C and 3D). Similar results were observed for the co-modification of ΔH4ac (Additional file 1, Figure S5). These results demonstrated that the level of co-modification in parallel gene pairs was higher than that of the divergent or convergent pairs. Moreover, parallel gene triplets (→ → →/← ← ←) showed greater similarity of acetylation change than the other directions, according to the composite profiles (Additional file 1, Figure S6). All of these results indicate that the coacetylation of neighboring genes might benefit more from the parallel transcriptional structure than the other two cases.

**Figure 4:**
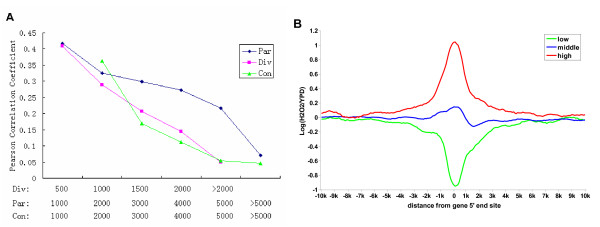
**The relationship between co-modification and gene distance**. (A) The correlation of ΔH3K14ac between immediate neighboring genes according to the gene 5' distance. Considering the effect of gene direction, the correlations were plotted according to the directions of pairs: parallel (Par), divergent (Div) and convergent (Con). (B) Composite profiles of ΔH3K14ac according to ΔH3K14ac level of the target gene in the translated region. All genes were assigned to 1 of 3 groups according to mean ΔH3K14ac in the coding regions (low, bottom 20%, middle, middle 60%; and high, top 20%), and aligned by the ORF 5'end start sites. From the start sites, the neighboring regions both upstream and downstream were segmented into 100 bins of 100 bp each by a moving window (size, 100 bp; step, 100 bp). The average ΔH3K14ac in each bin is plotted for each gene group.

To investigate the effect of gene distance on coacetylation, we assigned the immediate neighboring gene pairs to different groups according to their 5' distance and compared the level of co-modification between the groups. Considering the effect of gene directions, all pairs were first assigned to 1 of 3 groups according to their directions (parallel, divergent or convergent). We found that co-modification was inversely correlated to gene distance; i.e. it declined with increased 5' distance in all direction groups (Figure 4A). We plotted the composite profiles of ΔH3K14ac to explore the range of coacetylation with base-pair distance (Figure 4B). The profiles of different gene groups were indistinguishable in the regions beyond 5 kbp, meaning that coacetylation will occur within 5 kbp from the ORF 5' start site.

Taken together, our results show that the histone coacetylation in neighboring genes is highly correlated with both gene direction and distance.

### Gene expression is correlated with histone modifications of the neighboring 1 and 2 genes

**Figure 5:**
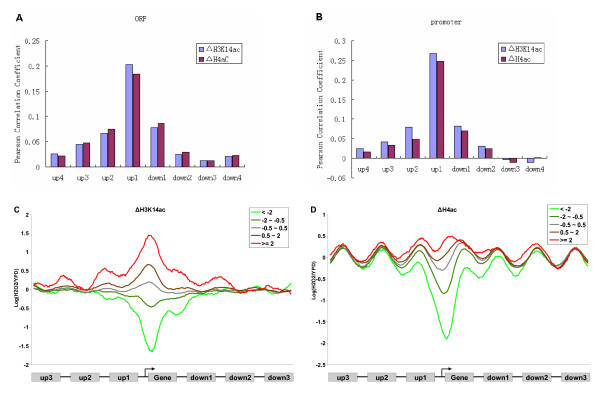
**The changes of gene expression correlated with the acetylation change of its neighboring genes**. (A) Correlation between change of gene expression and ΔH3K14ac (blue) and ΔH4ac (brown) of its neighbors in the coding regions. up1 ~ up4 means 4 linked genes upstream of the target gene; down1 ~ down4, 4 linked genes downstream. (B) Correlation between change of gene expression and the change of acetylation of its neighbors in promoter regions. (C) Composite profiles of ΔH3K14ac according to the change gene of expression. The genes were assigned to 1 of 5 groups according to their expression change under H_2_O_2_-stress conditions. (D) Composite profiles of H4ac change according to the change of expression.

The correlation between the change of a gene's expression [23] and the acetylation change of its neighboring genes was measured by the Pearson correlation coefficient across all genes in the genome. As shown in Figure 5A and 5B, the closest 2 genes upstream and the immediate neighboring genes downstream were significantly correlated (*p *< 0.001, for both translated and promoter regions; Table 1). The correlation with more distant genes was not significant (Table 1). We plotted the composite profiles of acetylation changes according to the change of gene expression to show the correlation in detail. The profiles showed clearly that the change of gene expression was correlated with the changes of acetylation in the gene region itself and in its neighboring 1 and 2 genes (Figure 5C and 5D). These results indicate that the co-modification has an effect on the coexpression of neighboring 1 or 2 genes.

**Table 1:** The correlation between expression change and acetylation change in neighboring genes

	**Correlation with neighboring ORFs**^**+**^	*p*-value*	Correlation with neighboring promoters^+^	*p*-value*
ΔH3K14ac				
up4	0.025	0.0655	0.026	0.0846
up3	0.042	0.0016	0.044	0.0035
up2	0.080	2.27E-06	0.066	2.51E-08
up1	0.267	2.77E-48	0.203	2.84E-81
down1	0.082	1.93E-08	0.078	6.39E-09
down2	0.031	0.0832	0.024	0.0300
down3	-0.003	0.3776	0.012	0.8123
down4	-0.011	0.1277	0.021	0.4523
				
ΔH4ac				
up4	0.016	0.1245	0.022	0.2631
up3	0.033	0.0008	0.047	0.0207
up2	0.049	7.87E-08	0.075	6.33E-04
up1	0.247	7.42E-40	0.184	1.32E-69
down1	0.070	5.11E-10	0.086	7.75E-07
down2	0.025	0.0379	0.029	0.0828
down3	-0.011	0.3898	0.012	0.4501
down4	0.002	0.1063	0.023	0.8984

+: the correlations were measured by the Pearson Correlation Coefficient between the gene expression change and the acetylation change of up4 ~ down4 gene in their ORFs and promoter regions, respectively.

*: *p*-value is the significant of the correlation coefficient by compared with the random.

It is noteworthy that the degree of correlation in the immediate upstream neighboring genes was much higher than that in the downstream immediate neighboring genes (Figure 5A and 5B), which might be attributed to the shorter distance between the upstream genes and the 5' end of the gene. When the 5' distance upstream and downstream was almost equal (e.g., the gene triplets were in all parallel structures i.e. (→ → →/← ← ←), the correlation with the downstream and the upstream gene had no significant difference (Additional file 1, Figure S7). Therefore, the 5' distance might play a key role in the effect of coacetylation on coexpression.

### Coacetylated domains in chromatin

**Figure 6:**
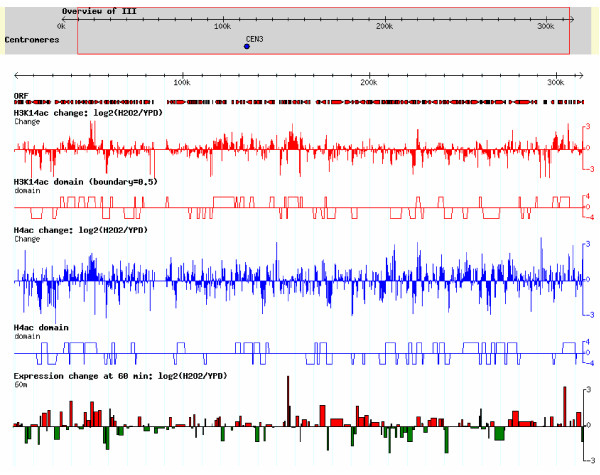
**Coacetylation domains in chromosome III**. The figure was generated by GBrowse (which is provided by GMOD: Genetic Model Organism Database; http://gmod.org). The tool can present the gene information in the genome and the user's data in the corresponding genomic locations. The top gray region in figure is the overview of chromosome III, and the coordinate indicates the location of the chromosome. The red arrows under "ORF" showed the locations and transcribed directions of genes. The red vertical line under H3K14ac change: log2(H2O2/YPD) and the blue line under H4ac change:log2(H2O2/YPD), respectively, show the change of H3K14ac and H4ac under H_2_O_2_-stress conditions in each probe. The red rectangles under H3K14ac domain (boundary 0.5) and the blue rectangles under H4ac domain indicate the domains with similar changes of H3K14ac and H4ac, respectively. The bottom subfigure is gene expression. The height and width of the rectangle indicate the degree of gene expression change and ORF length, respectively, and the color indicates up-regulated (red) or down-regulated (green).

**Figure 7:**
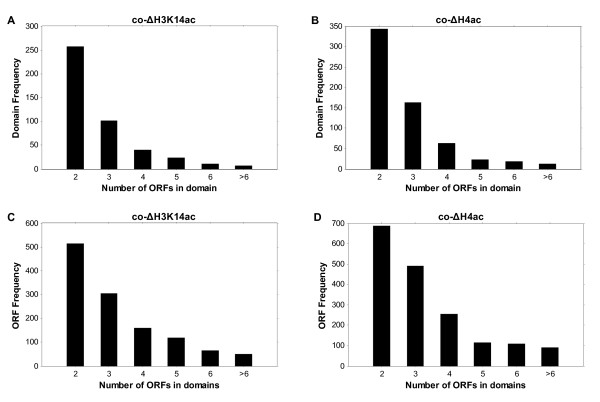
**The size distribution of coacetylation domains that covered at least 1 gene pair**. (A) A histogram of domain frequency according to the number of ORFs covered in the H3K14ac domains. (B) A histogram of domain frequency according to the number of ORFs covered in H4ac domains. (C) A histogram of ORF frequency according to the number of ORFs covered in H3K14ac domains. (D) A histogram of ORF frequency according to the number of ORFs covered in H4ac domains.

The autocorrelation analysis and composite profiles revealed the co-modification of neighboring genes from a global aspect, but they could not concretely tell which of the neighboring genes is/are co-modified. To find these coacetylated genes, we used a HMM to determine the continuous chromatin regions (i.e., coacetylated domains) that showed a similar change of acetylation (Methods). Coacetylated neighboring genes were shown to be within the same domains. An overview of the coacetylated domains in chromosome III is shown as a paradigm (Figure 6). There were 2081 co-ΔH3K14ac domains (similar change of H3K14ac) and 2305 co-ΔH4ac domains (similar change of H4ac) in the whole genome (Table 2). Over 20% of domains (co-ΔH3K14ac, 441/2081; co-ΔH4ac, 624/2305) covered 2 or more ORFs (Additional file 2) and 20 ~ 30% of genes in the whole genome showed changes of acetylation similar to that of their neighbors (1217 co-ΔH3K14ac genes and 1748 co-ΔH4ac genes; Table 2). The domains that included coacetylated genes were clusters of 2-4 genes (Figure 7). This result is in-line with the results of the autocorrelation analysis described above; i.e. that co-modification generally occurred within 1 or 2 neighboring genes.

**Table 2:** The number of coacetylated domains and coacetylated neighboring genes.

	all domains	domain size ≥2 genes	coacetylated genes	coacetylated pairs
co-ΔH3K14ac domain				
up-domain	725	208	524	474
down-domain	1356	233	693	896
totally	2081	441	1217	1370
				
co-ΔH4ac domain				
up-domain	963	356	1016	1235
down-domain	1342	268	732	794
totally	2305	624	1748	2029

### Gene pairs in coacetylation domains are coexpressed

**Figure 8:**
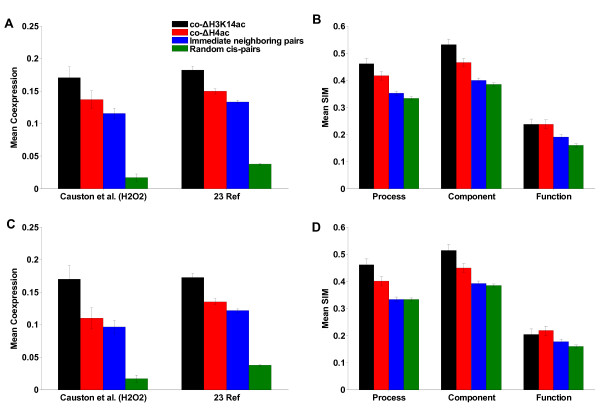
**Mean coexpression and mean functional similarity of gene pairs in coacetylation domains**. (A) Mean coexpression of gene pairs in co-ΔH3K14ac domains (black) and in co-ΔH4ac domains (red), immediate neighboring gene pairs (blue) and 10,000 random *cis*-pairs (green). The left-hand side is coexpression in H_2_O_2_-stress condition, and the right-hand side is the mean coexpression of data sets from 23 different references. (B) Mean semantic similarity (SIM) in Gene Ontology. (C) and (D) Mean coexpression and mean SIM of gene pairs by excluding the divergent pairs and pairs with the same TFBS.

We chose gene expression data under the conditions of H_2_O_2_-stress to be consistent with the histone acetylation experiments [23]. The coexpression of gene pairs was measured by the Pearson correlation coefficient of expression profiles between the two genes. After removing some special genome structures, including ORF-overlapping pairs and tandem duplicate pairs (Additional file 3), there were 1356 co-ΔH3K14ac pairs and 2010 co-ΔH4ac pairs. These co-modified pairs showed a significantly higher level of coexpression than random *cis*-pairs, which were defined as gene pairs within the same chromosomes (*p *< 10^-307^, Wilcoxon rank sum test, Figure 8A, left-hand side). The level of coexpression of co-ΔH3K14ac pairs was significantly higher than that of normal immediate neighboring pairs (*p *< 0.01, Wilcoxon rank sum test, Figure 8A, left-hand side).

To exclude the possibility of a higher level of coexpression under the hyperoxia stress condition being by chance alone, we collected the expression data from 23 different sources to repeat the analysis (Additional file 3). Gene pairs in coacetylated domains still showed a higher level of coexpression than random pairs (*p *< 10^-307^, Wilcoxon rank sum test) and normal immediate neighboring pairs (*p *< 0.01, Wilcoxon rank sum test, Figure 8A, right-hand side).

Sharing regulatory elements can lead to coexpression [1,9], so we excluded the immediate neighboring pairs of divergent transcription and gene pairs regulated by the same transcription factor (TF) from the coacetylated pairs (Additional file 3), which still showed a higher level of coexpression than random or normal immediate neighboring pairs (Figure 8C). These results indicate that co-modification is the sole contribution to the coexpression of neighboring genes.

**Figure 9:**
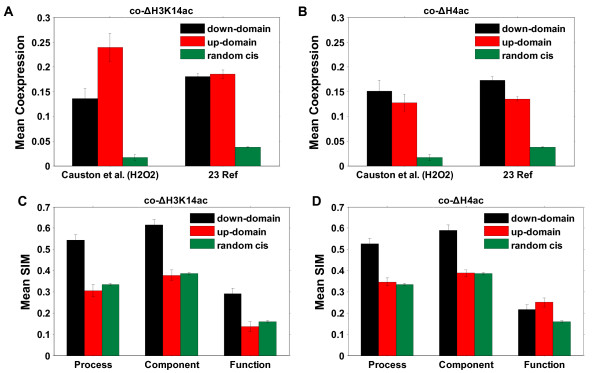
**The coexpression and co-function of gene pairs in up-domains and down-domains**. (A) Mean coexpression of gene pairs in co-ΔH3K14ac up-domains (red), down-domains (black) and random *cis*-pairs (green). (B) Mean coexpression of gene pairs in co-ΔH4ac up-domains (red), down-domains (black) and random *cis*-pairs (green). (C) Mean SIM of gene pairs in co-ΔH3K14ac up-domains (red), and down-domains (black) and random *cis*-pairs (green). (D) Mean SIM of gene pairs in co-ΔH4ac up-domains (red) and down-domains (black) and random *cis*-pairs (green).

According to our HMM method, there are two types of coacetylated domains, up-domains and down-domains (Methods). In the up-domains, acetylation in most probes was increased when conditions changed and the acetylation was decreased in down-domains. We computed the coexpression of gene pairs in up-domains and in down-domains to determine whether coacetylated pairs in the up-domains and those in the down-domains contributed equally to coexpression. Gene pairs in both domains showed a significantly higher level of coexpression than random *cis*-pairs (*p *< 10^-9^, Wilcoxon rank sum test; Figure 9A and 9B).

Taken together, these results demonstrate that coacetylated neighboring pairs are indeed coexpressed.

### Gene pairs in coacetylation domains share similar functions

Here, we used the method of semantic similarity (SIM) in Gene Ontology (GO) [24] to estimate the function similarity (co-function) of gene pairs (Additional file 3). The co-function was computed in three GO categories, Process, Component and Function. Similarly, ORF-overlapping and tandem duplicate pairs were excluded from the analysis. As shown in Figure 8B, coacetylated neighboring pairs had higher co-functions than random *cis*-pairs (*p *< 10^-5^, Kolmogorov-Smirnov (KS) test) and normal immediate neighboring pairs (*p *< 0.0001, KS test) in both GO Process and Component, but no significant difference was found in GO Function. After removing the divergent immediate neighboring pairs and gene pairs regulated by the same transcription factor (TF), coacetylated pairs still showed a higher level of co-function (for Process and Component, Figure 8D). However, the coacetylated pairs did not always contribute to the co-function. Gene pairs in down-domains showed a higher level of co-function than random *cis*-pairs (*p *< 10^-14^, KS test, for both GO Process and Component), but gene pairs in up-domains did not (Figure 9C and 9D), indicating that only pairs in down-domains contributed to the co-function of coacetylated neighboring pairs. The results of this study show that neighboring genes of similar modification can selectively share their functions and neighboring genes without similar functions can also be co-modified.

## Discussion

We have investigated the co-modification occurring in neighboring genes in the *S. cerevisiae *genome, and examined the relationship between co-modification, coexpression and co-function in neighboring gene pairs. The results show that neighboring genes indeed have similar histone modifications, and neighboring pairs of similar modifications exhibit similar expression.

In this study, autocorrelation analysis, composite profiles and HMM were used to explore the similarity of histone modification in neighboring genes. The results showed that histone modification in yeast exhibited a stringent similarity within only a small distance (neighboring 1 or 2 genes, or <10 kbp). These findings were quite different from those expected; i.e. that histone modification might have an effect on the coexpression of neighboring genes in a large range [1].

### Why the co-modification was restricted to a small range

According to the model proposed earlier, histone modification can generally occur within a region of about 100 kbp [1], which is much larger than the range of co-modification observed in this study. There are two possible reasons for the great difference between the results of these two studies.

First, we supposed that activation-associated modifications might have a different mechanism of spreading from silencing-associated modifications, and it might be distributed within only small local regions. Modification associated with silencing, such as H3K9me and H3K27me, could form long continuous domains in chromatin [25,26]. The model proposed earlier could well explain the spread of these modifications. However, the modifications analyzed in this study are associated mainly with activation, such as H3K9ac, H3K14ac and H3K4me3, etc. These modifications do not form long chromatin domains in yeast; therefore, a model of small-range spread might be more appropriate for the histone modification associated with activation.

Second, the interaction between different modifications could block the propagation of further modifications. Methylation of H3K36 has been found to inhibit acetylation in 3' coding regions [27,28], and dimethylation of H3K4 can lead to deacetylation in 5' transcribed regions [29]. In addition, our analysis showed that co-modification of H4ac in neighboring genes would significantly increase when its inhibitor, Set2 (which mediated H3K36me), was deleted from the yeast genome (Additional file 1, Figure S8). The inter-inhibition between histone modifications might impose stringent control of the distribution of modifications.

Coexpressed neighboring genes tend to be clustered within a narrow range. In yeast, highly coexpressed neighboring pairs and triplets, but not quadruplets, occur more frequently than expected by chance alone [10]. In mouse-ear cress (*Arabidopsis thaliana*), the local coexpression domains generally cover only 2-4 genes [30] or the chromatin regions within 12 kbp [31]. Fukuoka *et al. *[32] showed that the increased frequency of coexpression of neighboring genes is associated with chromatin within a short distance (< 10 kbp) in various eukaryotes. All of these findings show that the highly coexpressed neighboring genes occurred mainly within only a small region, which is consistent with the co-modification found in this study. Therefore, a small-range model of histone modifications appears to be more appropriate to explain the coexpression of neighboring genes and might be crucial for gene regulation. If activation-associated modifications could always spread over a long distance, many genes would be expressed inappropriately.

### Factors that might be responsible for co-modification of neighboring genes

The co-modification of neighboring genes is generally considered to be a result of common histone modification but several other factors might also lead to co-modification.

Sharing 5' regulatory elements or having similar regulatory elements, such as TFBS, might lead to co-modification. When TFs are bound to the gene promoter, histone-modifying enzymes recruited by the TFs might simultaneously change the modification of a gene and its neighbors that share the TFs. However, when neighboring pairs that shared TFs were included in our analysis, there were many neighboring genes with a similar change of acetylation. Moreover, although divergent pairs have a greater chance to share regulatory elements than parallel pairs, they do not exhibit a higher co-modification than parallel pairs. Therefore, although sharing regulator elements could lead to the co-modification of neighboring genes, it is far from being the main source.

All of the modification data in this study are normalized to the density of histone H3; therefore, the co-modification might be caused by histone H3. Indeed, we observed a similar change of H3 in neighboring genes (data not shown). However, there was little correlation between the change of acetylation and the change of H3 density across all probes in arrays (*r *< 0.02). Moreover, the change of H3 density did not show any correlation with gene expression change, but the acetylation change did (Additional file 1, Figure S9). Therefore, there is no evidence to support the possibility that normalization to H3 density can result in the co-modification of neighboring genes.

Systemic bias could be the cause of co-modification. One source of systemic bias has been found to strongly influence the coexpression of neighboring genes [12]. That is, when the probes of genes were printed in chips according to their position in the genome, regional bias around the chips could provide artifactual signals of coexpression [33,34]. When we examined the arrangement of probes in the chips that provided the modification data used in this study [21], we could not find any correlation between the location of probes in chips and their position in the genome. If there was a systemic bias induced by chips, all histone modifications and the control data should show the same result. However, H3K36me3, H3K4me1 and the control data did not show similarity in neighboring genes. Therefore, the co-modification found in our study cannot be attributed to the arrangement of probes in chips. To further test whether there was some unknown systemic bias, the autocorrelation analysis was applied to another histone modification data set [22] and gave the same results (Additional file 1, Figure S10). All of this evidence indicates that the co-modification found in this study cannot be attributed to systemic bias.

It is not clear whether other factors are involved in the co-modification but the factors we have identified, such as sharing regulatory elements, data normalization or systemic bias, are not the main causes. This indirectly supports the suggestion that similar histone modification of neighbors might play a key role in the co-modification of neighboring genes.

### The effect of gene direction on co-modification

In yeast, the earliest study of this issue found that divergent genes have a higher degree of coexpression than convergent genes [11]. However, a later study provided clear evidence that it is the 5' distance, not the gene directions, that results in the difference of coexpression in gene pairs with different transcription directions [10]. Unlike coexpression, co-modification in neighboring genes might be sensitive to the gene direction. In our study, after eliminating the difference of the 5' distance, the parallel gene pairs still showed a higher correlation than convergent or divergent pairs.

It is difficult to understand why the divergent pairs did not show more co-modifications because their promoters might be more likely to share the modifying enzymes. In fact, sharing regulatory elements encounters the same problem. Shared TFs were not significantly relevant to the coexpression of divergent pairs, although divergent pairs have a greater chance to share TFs [13]. We speculate that there might be some other mechanism that balances the effect of co-modification or sharing TFs on neighboring genes with different directions. For example, TFs might be orientation-dependent and regulate only 1 gene of a divergent pair [35], or some TFs could affect the transcription of genes that are not immediate downstream neighbors [10].

### The relationship between coexpression, co-modification and co-function

The functional meaning of coexpression between neighboring genes in yeast was investigated in earlier studies. Fukuoka *et al. *reported only that more than ten highly correlated neighboring pairs share the same GO function [32]. A more recent study showed that there is a difference from a random null model in the fraction of gene pairs in the same GO-slim process only for neighboring pairs with a high level of coexpression (*r *> 0.4) [12]. These studies indicated that, except for highly coexpressed neighboring pairs, most neighboring genes did not show similar functions.

In this study, immediate neighboring gene pairs did not show a higher level of co-function than random pairs (Figure 8B, *p *> 0.05, KS test, for all three GO categories) although they exhibited a higher level of coexpression than random pairs (Figure 8A, *p *< 10^-307^, Wilcoxon rank sum test, for both datasets from H_2_O_2_-stress and 23 references). The results indicate that coexpression in the neighboring genes is far from being a sufficient condition for their co-function. This conclusion is consistent with the finding that coexpression of linked genes in several mammalian genomes is generally disadvantageous [36]. Moreover, although the coacetylated gene pairs in both up-domains and down-domains had a higher level of coexpression than random pairs, only gene pairs in down-domains showed similar functions. This indicates that the coexpression driven by co-modification is independent of co-function.

We asked why gene pairs in the down-domains show similar functions but those in the up-domains do not. To address this question, the GO Term Finder (which was provided by Saccharomyces Genome Database i.e. SGD and was visited from http://db.yeastgenome.org/cgi-bin/GO/goTermFinder.pl) was used to find significant GO terms of genes in co-modification domains (Additional file 3). We found that genes in down-domains have many GO terms whose frequency of occurrence is significantly higher than the background (*p *< 0.01, provided by SGD). Many of them are ribosomal genes and participate in gene expression, ribosome biogenesis, translation etc. These genes tend to be clustered in the genome [32] and show similar functions. However, there are few GO terms in genes in up-domains. Some stress-responsive genes (i.e., induced by stress conditions), such as HSP10, HSP12, HSP30, HSP26, HSP104, MGA1, SYM1 and GRE3, were found in up-domains. The formation of up-domains might be associated with the genes that were activated under H_2_O_2_-stress conditions. However, these genes are not clustered in the genome and genes in up-domains do not show similar functions.

## Conclusions

This study, for the first time, used the autocorrelation analysis to investigate the similarity of histone modification between neighboring genes. We found that histone modifications H3K9ac, H4ac, H3K14ac, H3K4me2/3 and H3K9ac had similarities between neighboring genes. In contrast to expectation, these activation-associated modifications might be spread along the chromatin fiber within only a small region. Several hundred domains of similar acetylation changes covered more than one gene. Gene pairs in these domains showed high levels of coexpression in multiple data sets, but only pairs in the domains of increasing acetylation share similar functions. These findings suggest that a significant proportion of the coexpression of neighboring genes might be driven by the distribution of histone modification. The coexpression associated with co-modification, however, might be independent of the functional relationship of neighboring genes.

## Methods

### Data source and processing

The histone modification data was obtained from Pokholok *et al. *[21]. All of the histone modifications occupancies have been normalized to histone H3 density by the authors. We directly used the occupancy of H3K9ac, H3K14ac, H4ac and H3K4me3/me2/me1, H3K36me3, H3K79me3 and no-antibody control data in the analysis. The change of H3K14ac and H4ac were computed by log2(H2O2/YPD) for each probe if there was no missing value in both YPD and H_2_O_2_-stress conditions, and then normalized to a mean of zero and variance of 1 for all probes. The missing values (was few; 387 in ΔH3K14ac, 84 in ΔH4ac) were replaced by the mean value (i.e. zero). The data sets contained 41282 probes and 14138 of them were in intergenic regions and the other 27144 were located in coding regions. A total of 5526 ORFs were covered by the chips.

The gene expression data in hydrogen peroxide were obtained from Causton *et al. *[23]. The data in 0 minute were regarded as expression level in YPD conditions, and data in 60 minute were regarded as expression data in H_2_O_2_-stress conditions. The expression data sets contained 6115 genes after removing missing values. Expression change was defined as log2(H2O2/YPD) and normalized to mean of zero and variance of 1 for all genes. The other datasets used to compute the coexpression and the co-function were described in Additional file 3.

### The autocorrelation of histone modifications

As described above, the autocorrelation is generally a conception in signal processing. It can also test whether the observed value of a nominal, ordinal, or interval variable at one locality is independent of values of the variable at neighboring localities. We here used it to evaluate the similarity of histone modifications between a given gene series (for example, g_1_, g_2_, ..., g_n_; g_1_, g_2 _indicated the number of the gene according to genome order) and a lagged version (for example, g_2_, ..., g_n_, g_n+1_) of itself over successive gene intervals. The autocorrelation coefficient was calculated by the formula (1) and (2) when x = y.

(1) $$R{'}_{xy}(m)=\{\begin{array}{cc} \sum_{n=0}^{N-\left|m\right|-1}\left(x_{n+m}-\frac{1}{N}\sum_{i=0}^{N-1}x_{i}\right)\left(y_{n}-\frac{1}{N}\sum_{i=0}^{N-1}y_{i}\right) & m\geq 0\\ R{'}_{yx}\left(-m\right) & m<0 \end{array}$$

(2) $$R_{xy}(m)=R{'}_{xy}(m)/R'(0)$$

where *N *was the number of genes in a series and *m *was the gene interval, *x *and *y *were the gene series, *R*' was the cross-correlation between *x *and *y*, *R *was the cross-correlation coefficient when *R*' was normalized as 1 at zero lags (*m *= 0). When *x = y*, the *R *was the autocorrelation. The formulas were carried out by the function xcov (x, y, m,'coef') in the signal processing toolbox of Matlab.

The autocorrelation of the histone modifications was calculated by the following steps:

1. Pre-process the overlapping gene pairs and genes not covered by the probes. When the dubious gene, uncharacterized gene or pseudogene overlapped the verified genes, remove the former. When both pairs were the verified genes, remove the short one. When more than 5 continued linked genes were not covered by any probe, remove these genes. The remained genes which were not covered by the chips were replaced by a random gene. In this way, there were totally 17 long gene series in all 16 chromosomes (the chromosome XII was divided into two parts by a 19 continuous linked genes which were not covered by the chips, therefore, there were 17 long gene series), and these gene series totally contained 5855 non-overlapping genes.

2. Divide the long genes series into small part. The long gene series were divided into short gene series by a moving window (size, 50 genes; step, 1 gene; the reason was shown in the step 5). There were totally 5022 different short gene series with size as 50.

3. Calculate the autocorrelation for each short gene series. The histone modification level or change of a gene was defined as the average level of all probes in its translated or promoter region (which was defined as the intergenic region in upstream within 1 kbp), respectively. The autocorrelation for each short gene series was calculated by the formula (1) and (2).

4. Calculate the averaged autocorrelation across all short gene series. In each gene interval (i.e. the lag), the autocorrelations were summed up and divided the number of short gene series (i.e. 5022). The mean of autocorrelation was use to estimate the co-modification.

5. Determine the size of short gene series. We calculated the autocorrelation of H3K9ac for different sizes of short gene series in various gene intervals (Additional file 1, Figure S11). The mean autocorrelation was distinctly increasing with the increase of the size of short gene series when the size was less than 50. However, when the size was larger than 50, the autocorrelation in all intervals became flat. Thus, it was appropriate that the size of short gene series was taken as 50 genes. We also make the autocorrelation analysis for size as 100 and 200 and obtained the same results (data not shown).

### The Composite profiles

The composite profiles were firstly used by Pokholok *et al. *[21]. In our analysis, we extended the profiles to 7 continued linked ORFs. The ends of ORFs were defined at fixed points according to the position of translational start and stop sites. The length of each ORF was then subdivided into 40 bins of equal length, and probes were assigned according to their nearest corresponding relative position. Probes in intergenic regions between ORFs were similarly assigned following subdivision into 20 bins. The average histone modification enrichment (or change) for each subdivided bin was calculated. Thus, a profile with 400 bins was generated. The middle ORF was the target genes observed. All the target genes were normalized to the same transcription direction (**→**). When the target genes were assigned into different groups according to their histone modifications or expressions, the profile for each group was then created.

### Hidden Markov model algorithm

An automated method was required to find thousands of domains from genome-wide acetylation change data. Because of noise in the data, a naive threshold-based approach for determining domain position was highly inaccurate. Using sequences of noisy observed data, hidden Markov models are a powerful method for assigning probabilities to underlying hidden states. Hence, we developed an HMM approach that 1) allowed 4 types of domains: up, down, no-change and noisy domains, 2) allowed for variable-length of domains. The topology of our HMM was figured in Additional file 1, Figure S12.

The acetylation change for each probe was discretized into three different statuses: up, down and no-change according to boundary β (up, >β; down, < -β; no-change, [-β, β]). β was set to 0.5, so that, each of status had an approximately proportion of probe counts. The model parameters were estimated using EM algorithm with 5 iterations. The Viterbi algorithm was used to estimate the maximum likelihood state at each probe. All calculations were carried out using the software "Bayes Net Toolbox for Matlab" developed by Murphy http://bnt.googlecode.com/. Software will be downloadable, but not supported, on the web site of S.J.A. and G.C.Y.

The results were shown in Supplementary file 3. We discarded the noisy domains due to few numbers of them. Only the up-domains and down-domains were considered as the coacetylated domains.

### The statistical significance test

The co-modification and coexpression were tested by Wilcoxon rank sum test. And the co-function was tested by KS test. All of tests were carried out in Matlab. In these tests, if p was equal to zero, we showed as "*p *< 10^-307^", which was based on that the smallest positive number in Matlab is 2.2251 × 10^-308^.

## Authors' contributions

YD and XD designed the study, and YD also implemented the algorithms, analyzed the results and drafted the manuscript. XQ, CH, JW, JF and ZD participated in the analysis and discussion. All authors read and approved the final manuscript.

## Supplementary Material

Additional file 1 **The supplementary figures**. This file contained all of the supplementary figures (Additional file 1, Figure S1-S12) occurred in the paper. Click here for file

Additional file 2 **The coacetylated domains**. The information of coacetylated domains, including the range of each domains and contained genes. Click here for file

Additional file 3 **coexpression and co-function of gene pairs**. This file described how the coexpression and co-function were computed, which datasets were used and how the tandem duplicate pairs and pairs shared TFs were defined. Click here for file
