# Supplementary material for: Genome-wide analysis of the effect of histone modifications on the coexpression of neighboring genes in Saccharomyces cerevisiae
Source: BMC Genomics. 2010 Oct 9;11:550. doi: 10.1186/1471-2164-11-550 (PMC3091699; doi:10.1186/1471-2164-11-550)
Supplement: Additional file 1 — The supplementary figures. This file contained all of the supplementary figures (Additional file 1, Figure S1-S12) occurred in the paper. [file 1471-2164-11-550-S1.PDF]

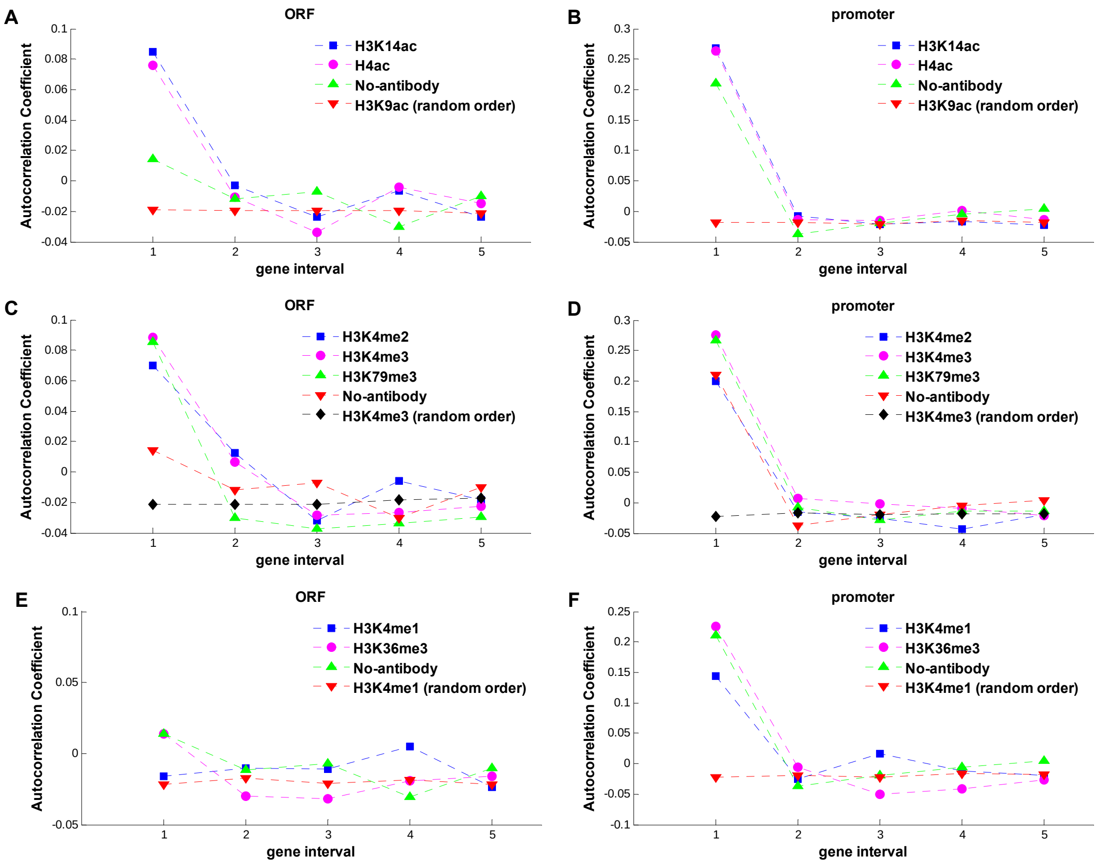

Figure S1. The mean of autocorrelation coefficient (AC) for histone acetylation and methylation within 5 gene intervals. The mean AC for (A and B) H3K14ac and H4ac (C and D) H3K4me2, H3K4me3 and H3K79me3 (E and F) H3K4me1 and H3K36me3 was compared with for no-antibody control data and for random order of genes. On the left-hand side (A, C and E), the modification level of a gene is defined as the average occupancy in its ORF region, and defined in its promoter region on the right-hand side (B, D and F).

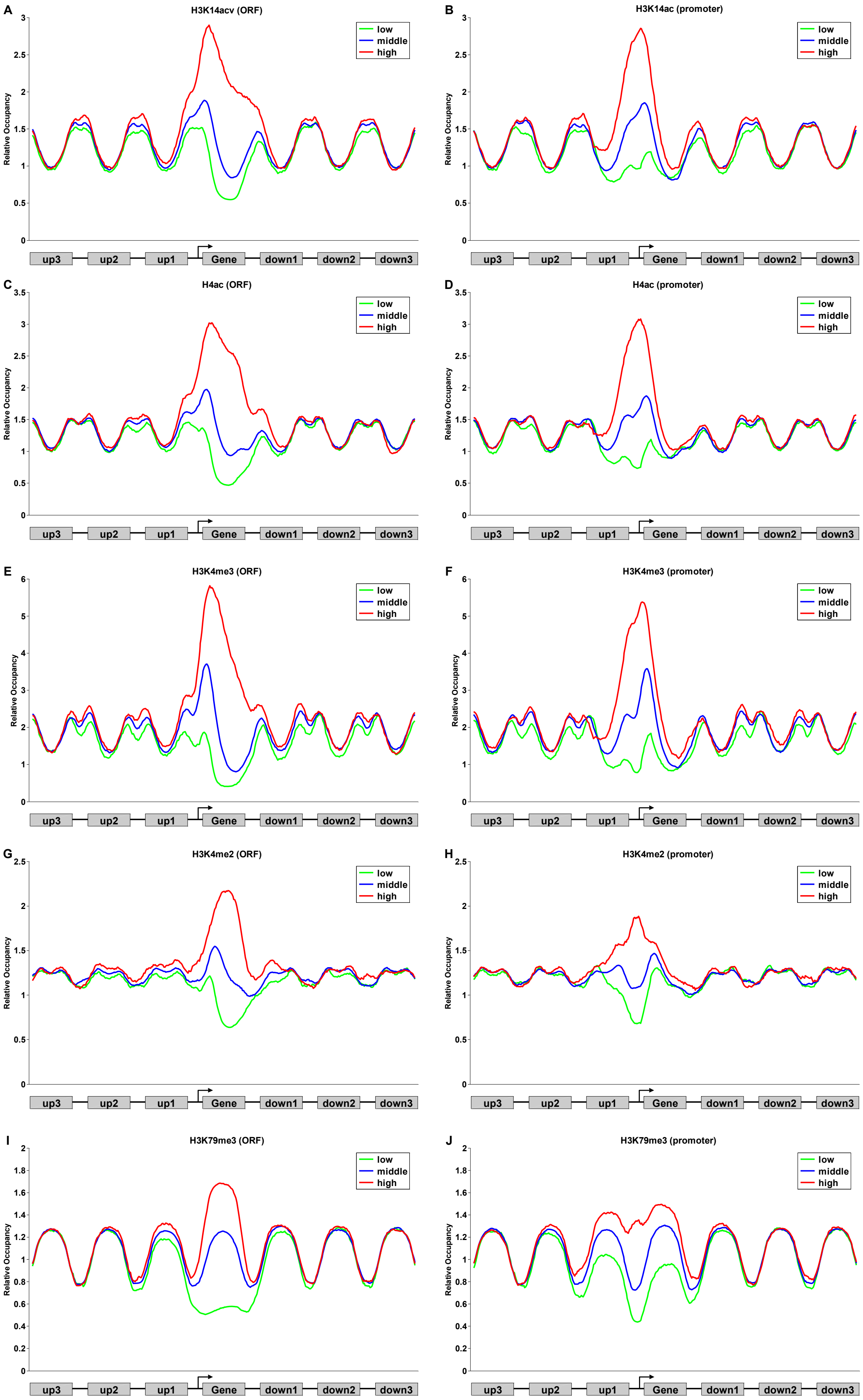
A
H3K14acv (ORF)
B
H3K14ac (promoter)
C
H4ac (ORF)
D
H4ac (promoter)
E
H3K4me3 (ORF)
F
H3K4me3 (promoter)
G
H3K4me2 (ORF)
H
H3K4me2 (promoter)
I
H3K79me3 (ORF)
J
H3K79me3 (promoter)
low
middle
high
Relative Occupancy
up3
up2
up1
Gene
down1
down2
down3

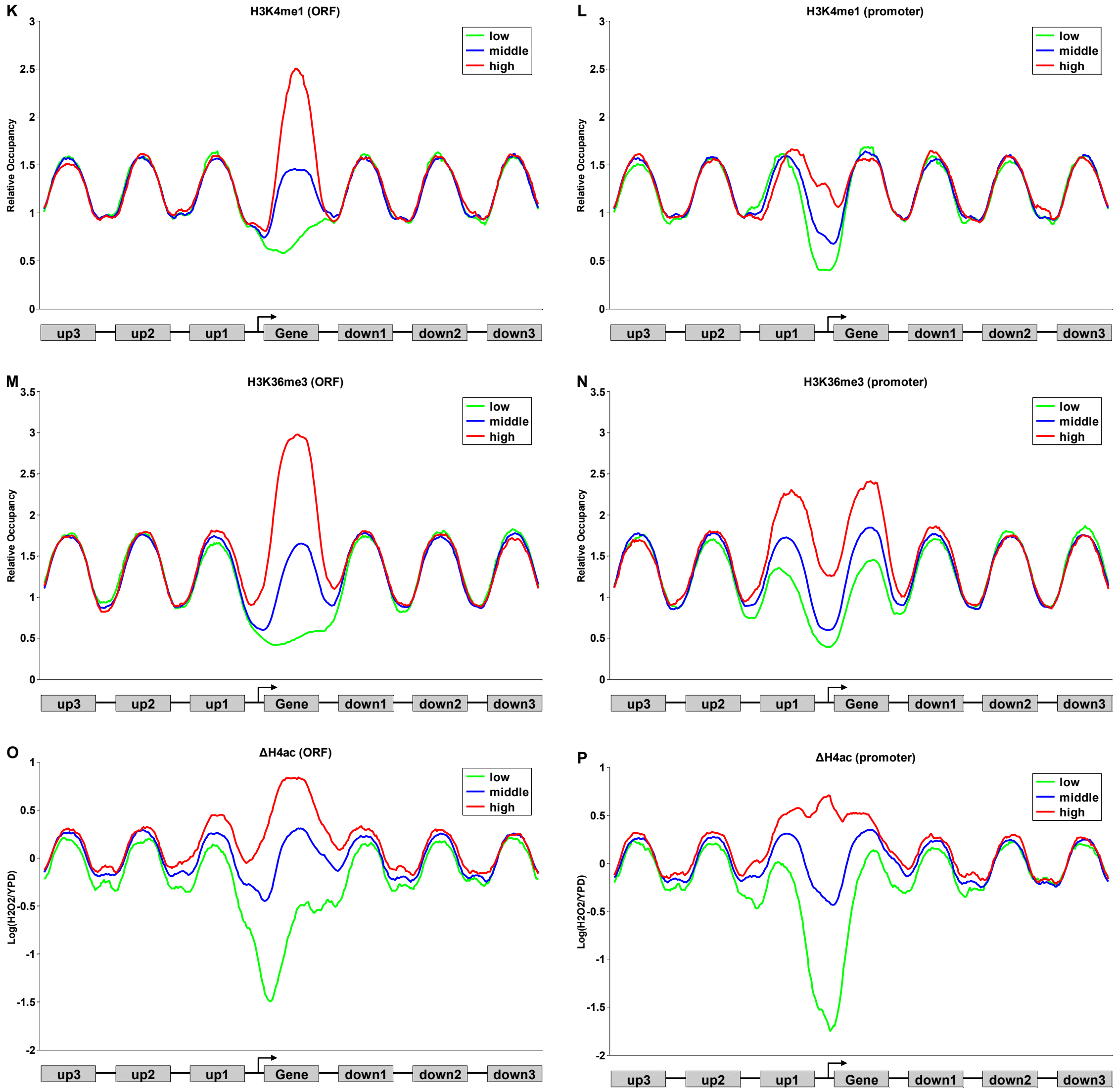

Figure S2. The composite profiles on an average gene and its neighbors.

The composite profiles of H3K14ac (A and B), H4ac (C and D), H3K4me3 (E and F), H3K4me2 (G and H), H3K79me3 (I and J), H3K4me1 (K and L), H3K36me3 (M and N) and ΔH4ac (O~P) were generated according to the mean level in the ORF regions (left-hand side) and promoter regions (right-hand side) of the target genes (**Gene**). The target genes were assigned to low (bottom 20%, green), middle (middle 60%, blue) or high (top 20%, red) groups according to the level of each histone modification and the composite profile was created for each group within 3

neighboring genes upstream and downstream.

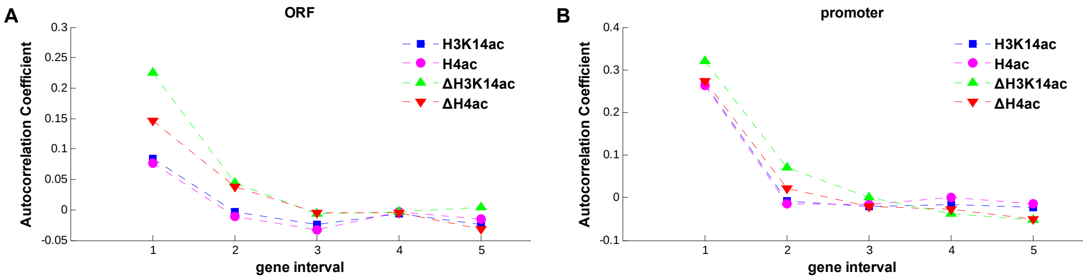

Figure S3. Comparing the autocorrelation between the histone acetylation occupancy and the change. (A) The mean autocorrelation of the H3K14ac occupancy (blue square) and H4ac occupancy (magenta circle), ΔH3K14ac (green up-triangle) and ΔH4ac (red down-triangle) in transcribed regions. (B) The mean autocorrelation in promoter regions. Details are as described for (A)

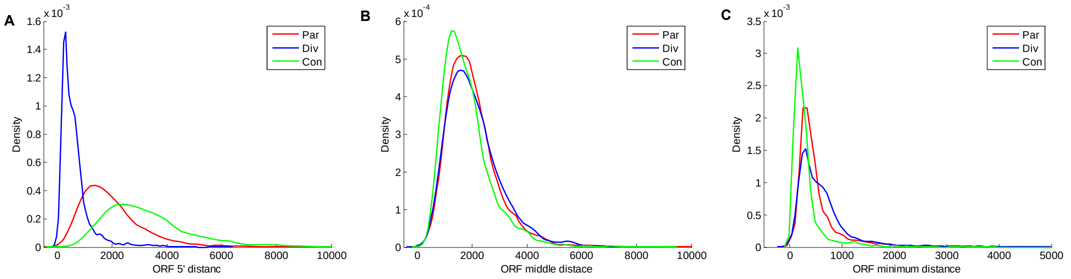

Figure S4. Distribution of distance of immediate neighboring gene pairs with different directions. (A) Distribution of ORF 5’ distance, which was defined as the distance between the translation start sites of ORFs. (B) Distribution of ORF middle distance, which was defined as the distance between the middle sites of ORFs. (C) Distribution of ORF minimum distance, which was defined as the distance between the closest sites of two ORFs. The gene distance of parallel, divergent and convergent pairs are shown in red, blue and green, respectively.

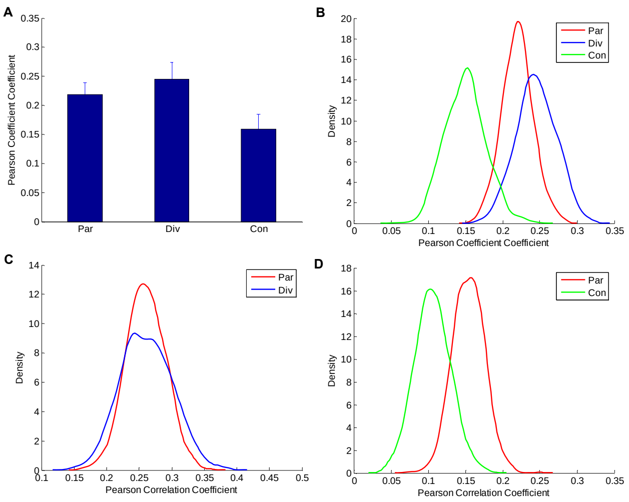

Figure S5. Comparing the correlation of ΔH4ac between immediate neighboring genes with different directions. The correlation was calculated by a bootstrap method (1000 times repeated) across neighboring pairs with different directions. The ΔH4ac level of a gene was defined as the average ΔH4ac in its ORF region. (A) The average correlation of ΔH4ac in divergent, parallel and convergent gene pairs. The error bar is the standard deviation. (B) The distribution of the correlation of ΔH4ac for divergent (red), parallel (blue) and convergent (green) gene pairs. (C) The distribution of correlation in ΔH4ac for parallel (red) and divergent (blue) pairs whose ORF 5' distances were 600~1200bp. (D) The distribution of correlation of ΔH4ac for parallel (red) and convergent (green) pairs whose ORF 5' distances were 1600~3600bp.

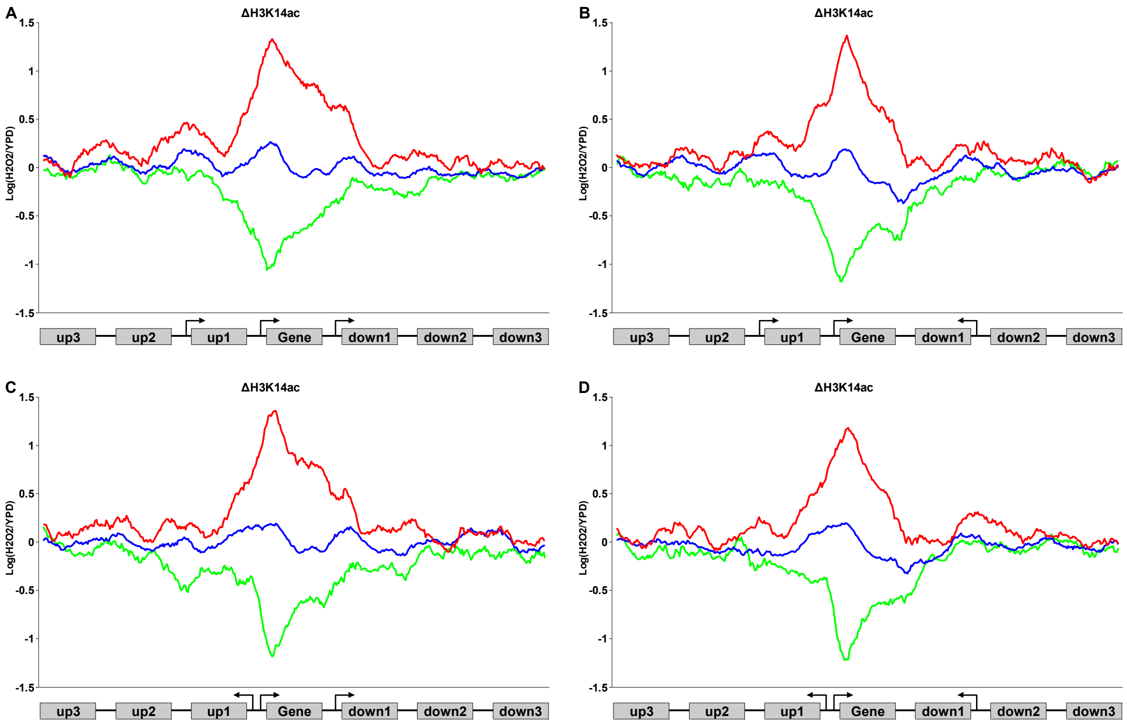

Figure S6. The composite profiles of ΔH3K14ac for gene triplets with different directions. The composite profiles were created according to the mean level of ΔH3K14ac of the target genes (Gene) in transcribed regions (low, bottom 20%, green; middle, middle 60%, blue; high, top 20%, red) for gene triplets of (A) →→→, (B) →→←, (C) ←→→ and (D) ←→← transcribed directions.

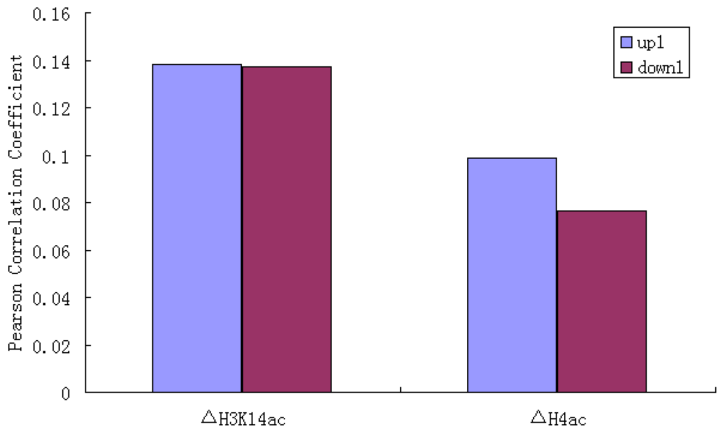

Figure S7. The correlation of between the expression change of gene and the histone acetylation change in the promoter of its neighboring genes when gene triplets are parallel transcribed (→→→).

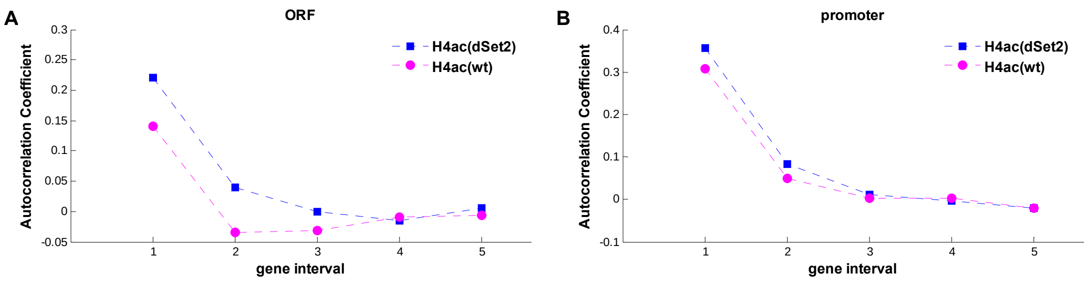

Figure S8. The autocorrelation coefficient (AC) for hyper-acetylation of H4 when Set2 was deleted or not deleted. The histone acetylation data are from Li et al. [1], who examined the hyper-acetylation of H4 (H4ac) in the wild type and in Set2 deletion mutants by using the same arrays as Pokholok et al. [2]. The AC for transcribed and promoter region is shown in A and B, respectively. The Set2 deletion mutants (blue square) had a higher AC than that in wild type (red circle) within 1 or 2 gene intervals.

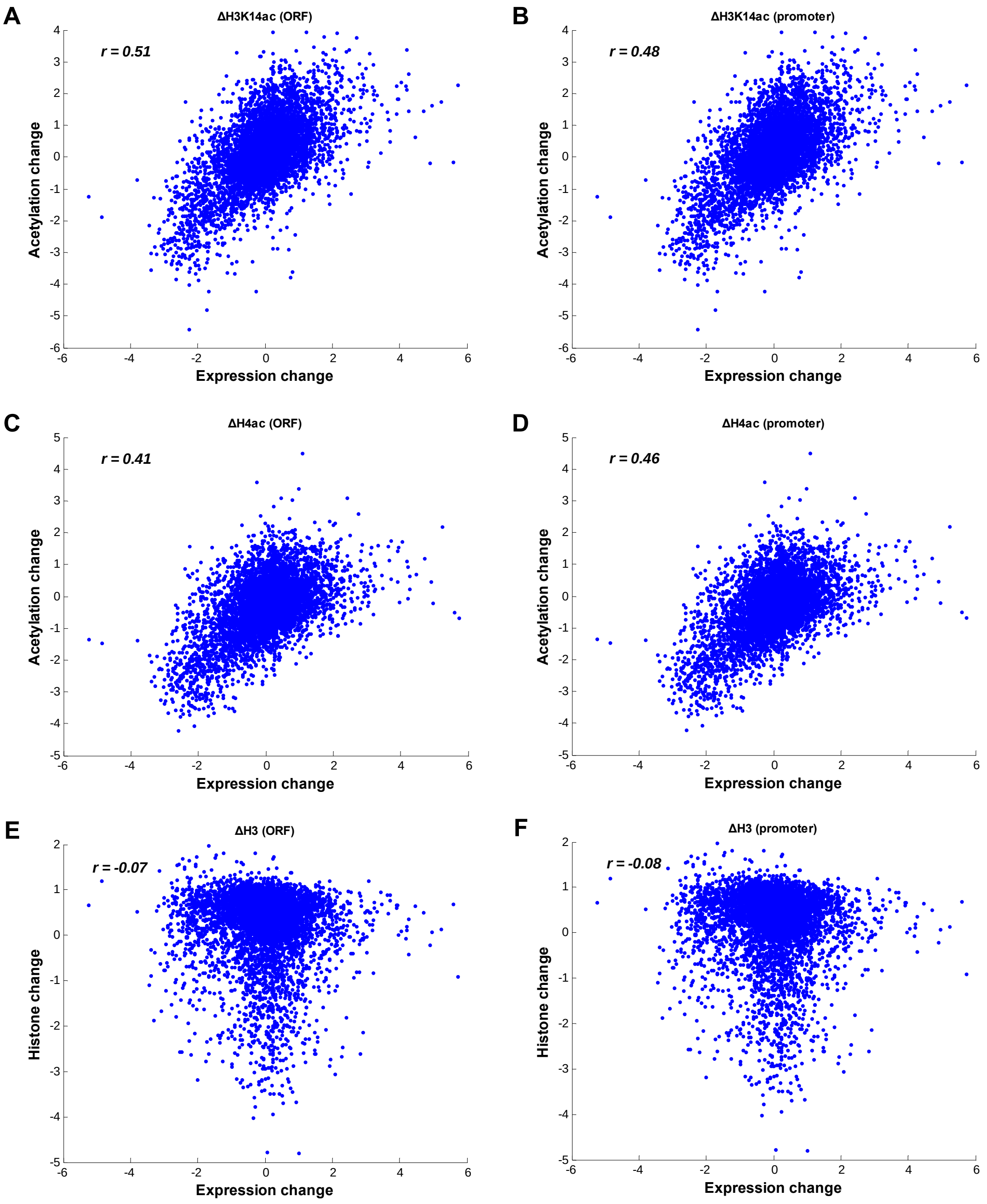

Figure S9. The correlation between expression change and the change of histone acetylation or histone density. The scatter map between expression change and ΔH3K14ac (A and B), ΔH4ac (C and D) and ΔH3 (E and F). On the left-hand side (A, C an E), the level of histone modification or histone density is defined as the mean level in translated regions (ORFs), and promoter regions on the right-hand side (B, D and F).

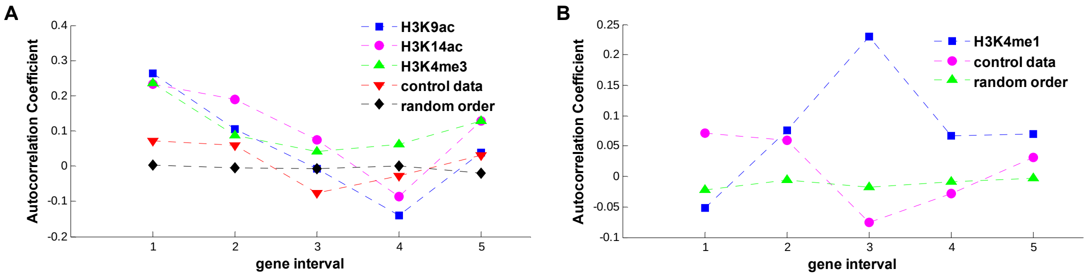

Figure S10. The autocorrelation of histone modification in Liu et al [3]. A total of 160 continued-linked and non-overlapping genes in chromosome III were divided into 61 gene series by a moving window (size 100 genes, step 1). The modification level was defined as the mean occupancy in ORF regions. The autocorrelation was calculated for each gene series, and the mean values are shown. (A) The autocorrelation of H3K9ac (blue square), H3K14ac (magenta circle) and H3K4me3 (green upward triangle) were compared with the control data (red downward triangle) from mock immunoprecipitations and H3K9ac for random gene order (dark diamond). H3K9ac, H3K14ac and H3K4me3 showed a significantly higher autocorrelation than the control data and random order when the gene interval was 1 ($p < 10^{-307}$, Wilcoxon rank sum test). In addition, H3K14ac showed a higher autocorrelation when the gene interval was 2 ($p < 10^{-307}$, Wilcoxon rank sum test). (B) The H3K4me1 did not show higher an autocorrelation higher than that for random gene order or control data within a 2 gene interval.

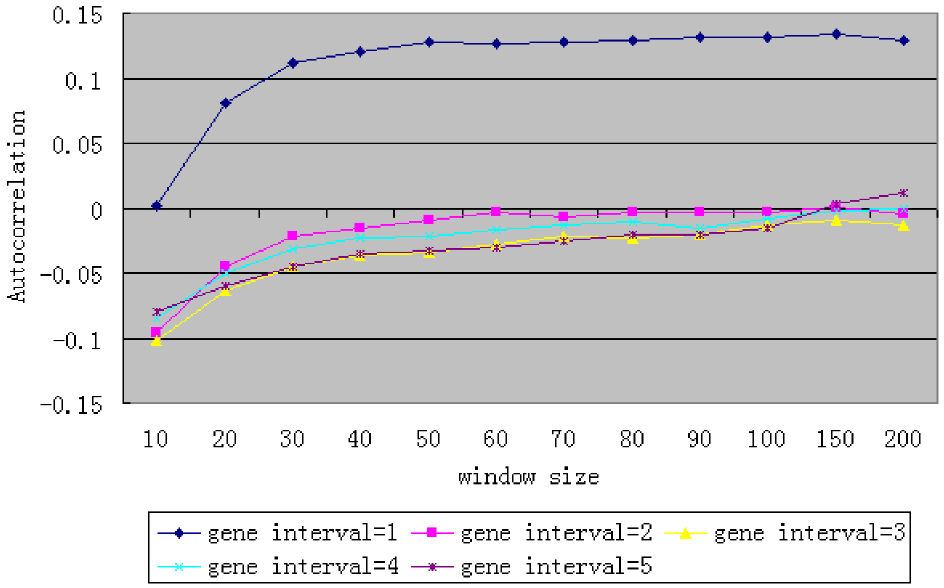

Figure S11. The relationship of the autocorrelation and the window size of the gene series. The mean autocorrelations of H3K9ac for windows size as 10, 20, 30, ...100, 150 and 200 are shown in each gene interval (≤5). When the window size was <50 genes, the autocorrelations profiles in each gene interval increased with the increasing window size. When the window size was ≥ 50 genes, the autocorrelation profiles in most intervals became flat.

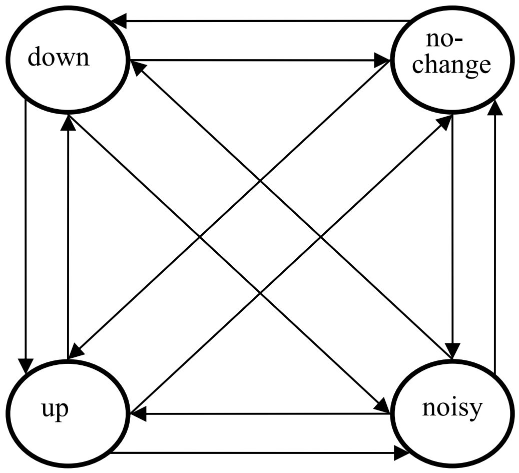

Figure S12. The topology of HMM. There were four statuses. The domains could be: down, most probes in the domains had a decrease of acetylation; up, most probes in the domains had an increase of histone acetylation; no-change, most probes had not significant change of acetylation; noisy, no significant enrichment of the probes with increasing, decreasing or no-change in the domains.

Reference

1. Li B, Gogol M, Carey M, Pattenden SG, Seidel C, Workman JL: **Infrequently transcribed long genes depend on the Set2/Rpd3S pathway for accurate transcription**. *Genes & Development* 2007, **21**(11):1422-1430.

2. Pokholok DK, Harbison CT, Levine S, Cole M, Hannett NM, Lee TI, Bell GW, Walker K, Rolfe PA, Herbolsheimer E *et al*: **Genome-wide Map of Nucleosome Acetylation and Methylation in Yeast**. *Cell* 2005, **122**(4):517-527.

3. Liu CL, Kaplan T, Kim M, Buratowski S, Schreiber SL, Friedman N, Rando OJ: **Single-Nucleosome Mapping of Histone Modifications in S. cerevisiae**. *PLoS Biology* 2005, **3**(10):e328.
