# Supplementary material for: Genome-wide analysis of the effect of histone modifications on the coexpression of neighboring genes in Saccharomyces cerevisiae
Source: BMC Genomics. 2010 Oct 9;11:550. doi: 10.1186/1471-2164-11-550 (PMC3091699; doi:10.1186/1471-2164-11-550)
Supplement: Additional file 3 — coexpression and co-function of gene pairs. This file described how the coexpression and co-function were computed, which datasets were used and how the tandem duplicate pairs and pairs shared TFs were defined. [file 1471-2164-11-550-S3.PDF]

Additional file 3 Coexpression and co-function of gene pairs

The coexpression in H_2O_2-stress conditions

The expression data were obtained from Causton *et al.*[1]. Six arrays in H_2O_2-stress conditions (including 0m, 10m, 20m, 40m 60m and 120m, 0m is YPD condition) were used in our analysis. The logarithmic ratio of expression between 10m (or 20m, 40m, 60m, and 120m) and 0m were normalized to 0 mean and standard deviation of 1. Coexpression of gene pairs was defined as the Pearson correlation coefficient the normalized expression data across 5 time points (10m ~ 120m).

The coexpression in 23 references

The information of 23 expression datasets was listed in Table S1. Expression data were normalized to 0 mean and standard deviation of 1, and missing data were replaced by the mean (i.e. 0). The coexpression was computed using Pearson Correlation Coefficient for each dataset. And for a given gene pairs the mean coexpression across 23 datasets was used.

Table S1 - 23 expression datasets used in this analysis.

Dataset	Array technology	Number of arrays
bae01 [2]	spot cDNA	20
cau01 [1]	Affymetrix	45
chi02 [3]	spot cDNA	20
cho98 [4]	Affymetrix	17
chu98 [5]	spot cDNA	7
coh02 [6]	spot cDNA	210
fle02 [7]	Affymetrix	30
gas00 [8]	spot cDNA	173

gas01 [9]	spot cDNA	52
hug00 [10]	spot cDNA	300
jel00 [11]	Affymetrix	25
mcc03 [12]	spot cDNA	15
mna04 [13]	spot oligonucleotides	291
mut01 [14]	spot cDNA	15
nat01 [15]	spot cDNA	10
nau02 [16]	spot cDNA	18
oro04 [17]	spot cDNA	133
rob00 [18]	spot cDNA	56
seg03 [19]	spot cDNA	36
spe98 [20]	spot cDNA	60
tu005 [21]	Affymetrix	36
yos02 [22]	spot cDNA	40
zhu00 [23]	spot cDNA	26

Tandem duplicated pairs

Tandem duplicate (TD) was defined as gene pairs within the same chromosomes and with protein sequences BLAST e-value <0.2. All yeast protein sequences downloaded from SGD (Saccharomyces Genome Database, ftp://genome-ftp.stanford.edu/pub/yeast/data_download/sequence/genomic_sequence/orf_protein/archive/orf_trans_all.20061110.fasta.gz) were compared using an all-and-all BLAST algorithm (its program was downloaded from NCBI: ftp://ftp.ncbi.nlm.nih.gov/blast/, the version was 2.2.15). Any pairs of genes of each other that showed sequence similarity (e-value <0.2) and were within same chromosomes was counted as a tandem duplicated pairs.

Gene pairs shared TFs

The TF-TG (TF: transcription factor; TG: target gene) interaction data were obtained

from Balaji et al. [24] who collected 157 TFs and 4410 TGs, and 12873 regulatory interactions. Neighboring gene pairs which were both interacted with any same TF were defined as pairs shared TFs.

Co-function of gene pairs

The co-functions of gene pairs were measured by the semantic similarity (SIM) in Gene Ontology (GO) which proposed by the Lord et al. [25]. This method is based on the information content and considered that a GO term in the ontology that is rather general containing less information than a term that is more specific and rare. We computed the SIM by referring to an application of this method in missing value estimation [26]. The GO ontology files (include Process, Component and Function files, version: 1.12) and gene association files (version: 1.1345) which required in this method were downloaded from GO website (http://www.geneontology.org/).

Reference

1. Causton HC, Ren B, Koh SS, Harbison CT, Kanin E, Jennings EG, Lee TI, True HL, Lander ES, Young RA: **Remodeling of Yeast Genome Expression in Response to Environmental Changes**. *Mol Biol Cell* 2001, **12**(2):323-337.
2. Baetz K, Moffat J, Haynes J, Chang M, Andrews B: **Transcriptional Coregulation by the Cell Integrity Mitogen-Activated Protein Kinase Slt2 and the Cell Cycle Regulator Swi4**. *Mol Cell Biol* 2001, **21**(19):6515-6528.
3. Chitikila C, Huisinga KL, Irvin JD, Basehoar AD, Pugh BF: **Interplay of TBP Inhibitors in Global Transcriptional Control**. *Molecular Cell* 2002, **10**(4):871-882.
4. Cho RJ, Campbell MJ, Winzeler EA, Steinmetz L, Conway A, Wodicka L, Wolfsberg TG, Gabrielian AE, Landsman D, Lockhart DJ *et al*: **A genome-wide transcriptional analysis of the mitotic cell cycle**. *Mol Cell* 1998, **2**(1): 65-73.
5. Chu S, DeRisi J, Eisen M, Mulholland J, Botstein D, Brown PO, I. H: **The transcriptional program of sporulation in budding yeast**. *Science* 1998, **282**(5389):699-705.
6. Cohen BA, Pilpel Y, Mitra RD, Church GM: **Discrimination between Paralogs using Microarray Analysis: Application to the Yap1p and Yap2p Transcriptional Networks**. *Mol Biol Cell* 2002, **13**(5):1608-1614.

7. Fleming JA, Lightcap ES, Sadis S, Thoroddsen V, Bulawa CE, Blackman RK: **Complementary whole-genome technologies reveal the cellular response to proteasome inhibition by PS-341**. *Proceedings of the National Academy of Sciences of the United States of America* 2002, **99**(3):1461-1466.
8. Gasch AP, Spellman PT, Kao CM, Carmel-Harel O, Eisen MB, Storz G, Botstein D, Brown PO: **Genomic Expression Programs in the Response of Yeast Cells to Environmental Changes**. *Mol Biol Cell* 2000, **11**(12):4241-4257.
9. Gasch AP, Huang M, Metzner S, Botstein D, Elledge SJ, Brown PO: **Genomic Expression Responses to DNA-damaging Agents and the Regulatory Role of the Yeast ATR Homolog Mec1p**. *Mol Biol Cell* 2001, **12**(10):2987-3003.
10. Hughes TR, Marton MJ, Jones AR, Roberts CJ, Stoughton R, Armour CD, Bennett HA, Coffey E, Dai H, He YD *et al*: **Functional Discovery via a Compendium of Expression Profiles**. *Cell* 2000, **102**(1):109-126.
11. Jelinsky SA, Estep P, Church GM, Samson LD: **Regulatory Networks Revealed by Transcriptional Profiling of Damaged Saccharomyces cerevisiae Cells: Rpn4 Links Base Excision Repair with Proteasomes**. *Mol Cell Biol* 2000, **20**(21):8157-8167.
12. McCammon MT, Epstein CB, Przybyla-Zawislak B, McAlister-Henn L, Butow RA: **Global Transcription Analysis of Krebs Tricarboxylic Acid Cycle Mutants Reveals an Alternating Pattern of Gene Expression and Effects on Hypoxic and Oxidative Genes**. *Mol Biol Cell* 2003, **14**(3):958-972.
13. Mnaimneh S, Davierwala AP, Haynes J, Moffat J, Peng W-T, Zhang W, Yang X, Pootoolal J, Chua G, Lopez A *et al*: **Exploration of Essential Gene Functions via Titratable Promoter Alleles**. *Cell* 2004, **118**(1):31-44.
14. Mutka SC, Walter P: **Multifaceted Physiological Response Allows Yeast to Adapt to the Loss of the Signal Recognition Particle-dependent Protein-targeting Pathway**. *Mol Biol Cell* 2001, **12**(3):577-588.
15. Natarajan K, Meyer MR, Jackson BM, Slade D, Roberts C, Hinnebusch AG, Marton MJ: **Transcriptional Profiling Shows that Gcn4p Is a Master Regulator of Gene Expression during Amino Acid Starvation in Yeast**. *Mol Cell Biol* 2001, **21**(13):4347-4368.
16. Nautiyal S, DeRisi JL, Blackburn EH: **The genome-wide expression response to telomerase deletion in Saccharomyces cerevisiae**. *Proceedings of the National Academy of Sciences of the United States of America* 2002, **99**(14):9316-9321.
17. O'Rourke SM, Herskowitz I: **Unique and Redundant Roles for HOG MAPK Pathway Components as Revealed by Whole-Genome Expression Analysis**. *Mol Biol Cell* 2004, **15**(2):532-542.
18. Roberts CJ, Nelson B, Marton MJ, Stoughton R, Meyer MR, Bennett HA, He YD, Dai H, Walker WL, Hughes TR *et al*: **Signaling and circuitry of multiple MAPK pathways revealed by a matrix of global gene expression profiles**. *Science* 2000, **287**(5454):873-880.
19. Segal E, Shapira M, Regev A, Pe'er D, Botstein D, Koller D, Friedman N: **Module networks: identifying regulatory modules and their condition-specific regulators from gene expression data**. *Nat Genet* 2003, **34**(2):166-176.
20. Spellman PT, Sherlock G, Zhang MQ, Iyer VR, Anders K, Eisen MB, Brown PO, Botstein D, Futcher B: **Comprehensive Identification of Cell Cycle-regulated Genes of the Yeast Saccharomyces cerevisiae by Microarray Hybridization**. *Mol Biol Cell* 1998,

9(12):3273-3297.

21. Tu BP, Kudlicki A, Rowicka M, SL M: **Logic of the yeast metabolic cycle: temporal compartmentalization of cellular processes**. *Science* 2005, **310**(5751):1152-1158.
22. Yoshimoto H, Saltsman K, Gasch AP, Li HX, Ogawa N, Botstein D, Brown PO, Cyert MS: **Genome-wide Analysis of Gene Expression Regulated by the Calcineurin/Crz1p Signaling Pathway in Saccharomyces cerevisiae**. *J Biol Chem* 2002, **277**(34):31079-31088.
23. Zhu G, Spellman PT, Volpe T, Brown PO, Botstein D, Davis TN, Futcher B: **Two yeast forkhead genes regulate the cell cycle and pseudohyphal growth**. *Nature* 2000, **406**(6791):90-94.
24. Balaji S, Babu MM, Iyer LM, Luscombe NM, Aravind L: **Comprehensive Analysis of Combinatorial Regulation using the Transcriptional Regulatory Network of Yeast**. *Journal of Molecular Biology* 2006, **360**(1):213-227.
25. Lord PW, Stevens RD, Brass A, Goble CA: **Investigating semantic similarity measures across the Gene Ontology: the relationship between sequence and annotation**. *Bioinformatics* 2003, **19**(10):1275-1283.
26. Tuikkala J, Elo L, Nevalainen OS, Aittokallio T: **Improving missing value estimation in microarray data with gene ontology**. *Bioinformatics* 2006, **22**(5):566-572.
